# Outcomes, Interventions and Funding in Randomised Research Published in High-Impact Journals

**DOI:** 10.1186/s13063-018-2978-8

**Published:** 2018-10-29

**Authors:** Patrick Grey, Andrew Grey, Mark J. Bolland

**Affiliations:** Department of Medicine, University of Auckland, Private Bag 92024, Auckland, New Zealand

**Keywords:** Randomised clinical trials, Outcomes, Surrogate, Interventions, Sponsorship

## Abstract

**Background:**

Randomised clinical trials are pivotal in guiding clinical practice. Trials with surrogate outcomes and industry sponsorship might be less reliable than those with hard outcomes and independent sponsorship. The types of interventions evaluated in randomised clinical trials might not reflect the diversity of those employed in clinical practice.

**Methods:**

We assessed the types of primary outcome, types of intervention and sponsorship of 360 randomised clinical trials evaluating 416 interventions, published in seven major general medical journals and 10 speciality medical journals in five clinical disciplines.

**Results:**

Primary outcomes were surrogate in 223/360 (62%) trials. Neither type of journal nor source of sponsorship was associated with type of primary outcome. Among the interventions evaluated, 233/416 (56%) were drugs, 17/416 (4%) devices and 49/416 (12%) procedures. The majority of trials were non-industry funded (220/360, 61%). Trials of drug interventions and those with industry sponsorship were more common in specialty than general journals (68% vs 48% and 55% vs 25%, respectively). Industry sponsorship was not associated with results for the primary outcome but was strongly associated with trials of drugs and devices.

Within the groups of both general and speciality journals, there were wide ranges in the prevalence of industry funding (7–63% and 37–70%, respectively), but in all cases the prevalence of hard primary outcomes was <40%.

**Conclusions:**

Most randomised clinical trials published in influential journals reported surrogate primary outcomes and assessed drug interventions. Trials that evaluated devices and procedures were infrequently published, despite the prominence of each type of intervention in clinical practice. Industry funding was more common for trials published in specialty than general journals but was not associated with more positive results for primary outcomes or with a greater preponderance of surrogate outcomes.

## Background

Randomised trials are the highest level of evidence in clinical research and are, therefore, pivotal in guiding clinical practice [1]. Accordingly, such trials are often published in prestigious high-impact journals. However, some randomised trials may be less robust than others. Trials with end points that are surrogates for clinically important (hard) outcomes may be less reliable because beneficial changes in surrogate end points do not necessarily translate to clinically meaningful benefits [2]. For example, a decrease in glycated haemoglobin, a surrogate end point frequently reported in trials of type 2 diabetes, is not invariably accompanied by reductions in clinical events such as incidence of diabetes complications or vascular events [3]. In oncology, only 5 of 23 drugs that obtained regulatory approval based on trials with surrogate end points between 2008 and 2012 and that were subsequently evaluated in trials with hard primary outcomes were found to confer a survival benefit [4]. There are numerous examples of treatments that entered clinical practice based on favourable effects on surrogate end points but which were subsequently found to be harmful [2].

The sponsorship of clinical trials might also influence their reliability. Systematic reviews indicate that industry-sponsored drug research is more likely to report beneficial effects and favourable safety profiles than non-industry-sponsored drug research [5, 6]. Research on new drugs and devices is almost exclusively sponsored by industry, and almost always includes the conduct of randomised trials with surrogate end points. The thrust of biomedicine to produce new and improved interventions might favour the reporting of clinical trials of new treatments in prestigious influential journals [7], particularly if those journals profit from such publications [8].

Clinical practice comprises a range of management strategies. Interventions include medications, medical devices and technologies, and procedures. The relative proportions of these interventions that are applied varies by discipline, but devices and procedures are an integral part of the working lives of most practitioners, and their use affects patient outcomes. Concerns have been expressed that medical devices can enter clinical practice without supporting evidence from clinical trials [9–11], perhaps because there are less stringent regulations for them than for pharmaceuticals [12].

In the current work, we set out to determine the prevalence of surrogate primary outcomes in clinical trials published in high-impact journals, the prevalence of the types of intervention assessed in such trials and whether there is a higher proportion of surrogate primary outcomes in industry-sponsored trials than non-industry-funded trials. Accordingly, we assessed the types of primary end points, types of intervention assessed and sponsorship in a set of randomised trials published in major internal medicine and specialty journals.

## Methods

### Dataset of clinical trials

We assessed randomised clinical trials published in 7 general and 10 specialty journals in 2013 and 2014. We focused on the journals that are likely to have the greatest readership and therefore, are most likely to influence clinical practice. The general medical journals were those with the highest impact factors: *The New England Journal of Medicine* (*NEJM*), *Lancet*, *Journal of the American Medical Association* (*JAMA*), *The  British Medical Journal*, *JAMA Internal Medicine*, *Annals of Internal Medicine* and *PLoS Medicine*. The specialty journals were the two journals with the highest impact factors that publish clinical research in each of five disciplines. For cardiology, we assessed publications in *Journal of the American College of Cardiology* and *Circulation*, for infectious diseases those in *Lancet Infectious Diseases* and *Clinical Infectious Diseases*, for neurology those in *Lancet Neurology* and *Neurology*, for oncology *Lancet Oncology* and *Journal of Clinical Oncology*, and for respiratory medicine *Lancet Respiratory Medicine* and *American Journal of Respiratory and Critical Care Medicine*.

**Fig. 1:**
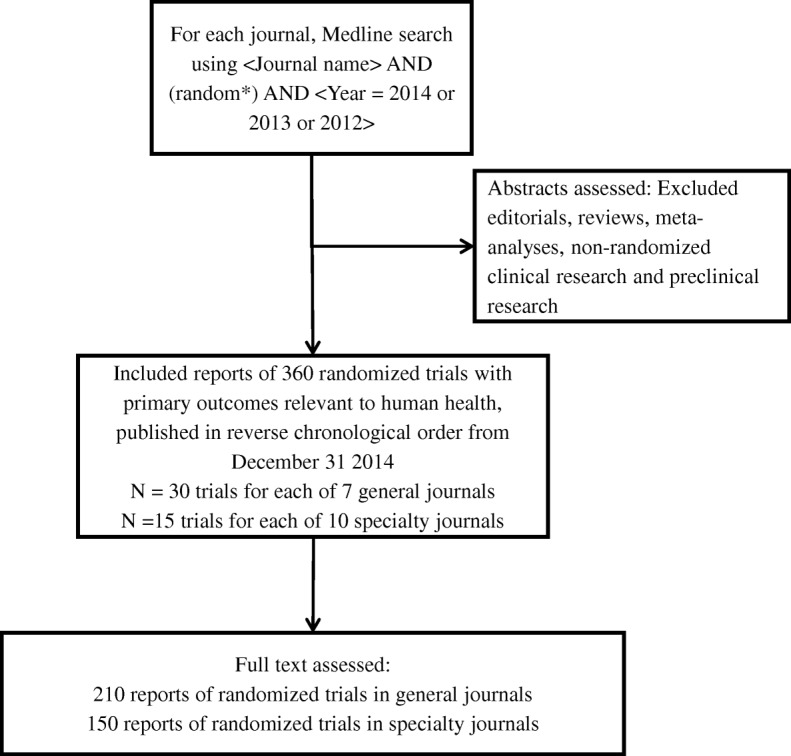
Collation of reports of randomised trials

One investigator (PG) searched Medline for reports of randomised clinical trials in each journal, collating the most recent publications before 31 December 2014 (Fig. 1). For each general medical journal, 30 publications were assessed (*n* = 210 in total) and 15 were assessed for each specialty journal (*n* = 30 for each speciality, *n* = 150 in total). The number of publications assessed was determined pragmatically. A second investigator (AG) checked the search and collation of publications.

We included all randomised trials conducted in humans, with primary outcomes relevant to human health. We excluded meta-analyses. To determine the eligibility of a publication, we reviewed its abstract and, if necessary, the full text.

### Outcomes

For each included publication, we reviewed the full text of the trial report. We extracted data on trial sponsorship, primary outcome(s), trial design, type of intervention and trial results. All data were extracted by one investigator (PG) and checked by a second investigator (AG). In the event of disagreement, a third investigator (MB) provided an assessment and the majority view was recorded.

We categorised trial sponsorship as any industry (solely industry funding or mixed industry/non-industry funding) or non-industry. Primary outcomes of the trials were categorised as hard, surrogate or mixed, and single, co-primary or composite. We used the Institute of Medicine definition of surrogate outcomes as “biomarker[s] intended to substitute for a clinical endpoint [and] expected to predict clinical benefit (or harm …) based on epidemiologic, therapeutic, pathophysiologic, or other scientific evidence” [13]. Hard outcomes are patient-important end points that are definitive with respect to the disease process, and reflect how a patient feels, functions or survives [13]. Table 1 sets out examples from the current analysis of hard outcomes reported in trials in speciality journals. Interventions were categorised as drugs, devices, procedures or other. The latter category included interventions such as strategies for delivery of care, application of health systems or public health initiatives, health education, and counselling. Trial results for each intervention were categorised regarding the primary trial outcome as beneficial, neutral or harmful, based upon statistical assessments in the publication. All assessments were initially performed by one investigator (PG) and checked by a second (AG). In the event of disagreement, a third investigator (MB) provided an assessment and the majority view was recorded.

**Table 1:** Examples of hard outcomes (context) in trials reported in speciality journals

Neurology	Headache days (migraine) Incident clinical disease (multiple sclerosis) Living circumstances (stroke) Survival (amyotrophic lateral sclerosis)
Oncology	Survival (various cancers) Incident disease (breast cancer)
Cardiology	Myocardial infarction (various interventions) Stroke (various interventions) Mortality (various interventions) Incident disease (atrial fibrillation)
Respiratory medicine	Incident clinical disease (asthma, chronic obstructive pulmonary disease, pneumonia) Abstinence (cigarette smoking)
Infectious Diseases	Clinical resolution of infection (pneumonia, urogenital infections) Incident clinical disease (soft tissue infections, malaria) Clinical adverse events (vaccinations)

### Statistics

We used descriptive statistics to report the prevalence of types of sponsorship, primary outcome, trial design and trial results, and chi squared or Fisher’s exact test to compare categorical variables between trials with different types of sponsorship and in different types of journal.

## Results

We assessed 360 trials that collectively reported the effects of 416 interventions (Table 2). We found that 233/416 interventions (56%) were drugs, 17/416 (4%) were devices and 49/416 (12%) were procedures. Primary outcomes were single in 237/360 trials (66%), co-primary in 47/360 trials (13%) and composite in 76/360 trials (21%). Primary outcomes were surrogate in 223/360 (61%) trials. A further 50 trials reported mixed surrogate/hard primary outcomes, meaning that in only 87/360 (24%) trials was the primary outcome hard. The primary outcome was clearly stated in the abstract of 286/360 (79%) of trial publications. The majority of trials were non-industry funded (220/360, 61%).

**Table 2:** Trial characteristics according to type of journal of publication

	All journals	General journals	Specialty journals	*P* ^*^
Trials (*n*) Interventions (*n*)	360 416	210 249	150 167	
Intervention				
Drug	233 (56%)	120 (48%)	113 (68%)	< 0.0001
Device	17 (4%)	9 (4%)	8 (5%)	
Procedure	49 (12%)	29 (12%)	20 (12%)	
Other	117 (28%)	91 (37%)	26 (16%)	
Primary outcome				
Single	237 (66%)	135 (64%)	102 (68%)	0.006
Co-primary	47 (13%)	37 (18%)	10 (7%)	
Composite	76 (21%)	38 (18%)	38 (25%)	
Surrogate	223 (62%)	131 (62%)	92 (61%)	0.90
Mixed	50 (14%)	30 (14%)	20 (13%)	
Hard	87 (24%)	49 (23%)	38 (25%)	
Clearly stated in abstract	286 (79%)	179 (85%)	107 (71%)	0.002
Funding				
Any industry	136 (38%)	53 (25%)	83 (55%)	< 0.001
Non-industry	220 (61%)	156 (74%)	64 (43%)	
Not stated	4 (1%)	1 (< 1%)	3 (2%)	

^*^When comparing general journals vs speciality journals

Table 2 also shows the trial characteristics according to the type of journal. A greater proportion of trials studying interventions other than drugs, devices and procedures were published in general journals than specialty journals. Similar proportions of trials with solely surrogate primary end points were published in general and specialty journals (62% and 61%, respectively).

A higher proportion of trials published in specialty journals than in general journals had industry funding (83/150, 55% vs 53/210, 25%) (*P* < 0.001). In specialty journals, 67/150 (45%) trials were industry funded and included a drug intervention, compared to 37/210 (18%) in general journals (*P* < 0.001). In specialty journals, higher proportions of trials with both surrogate outcomes (61/111, 55%) and hard outcomes (22/36, 61%) had industry funding than those in general journals (39/161, 24% and 14/48, 29%, respectively, *P* < 0.01 for each comparison).

Table 3 shows the trial characteristics according to source of funding. Trials with interventions other than drugs, devices and procedures were more frequently sponsored by non-industry sources. No differences were observed in types of primary outcomes, or results for primary outcomes between trials with any industry funding and trials with no industry funding (Table 3).

**Table 3:** Trial characteristics by funding source

	Any industry funding	No industry funding	*P*
Trials (*n*)^a^ Interventions (*n*)	136 149	220 262	
Intervention			
Drug	115 (77%)	114 (44%)	< 0.0001
Device	13 (9%)	4 (2%)	
Procedure	12 (8%)	36 (14%)	
Other	9 (6%)	108 (41%)	
Primary outcome			
Single	91 (67%)	143 (65%)	0.76
Co-primary	26 (19%)	49 (22%)	
Composite	19 (14%)	28 (13%)	
Surrogate	77 (57%)	145 (66%)	0.20
Mixed	23 (17%)	27 (12%)	
Hard	36 (26%)	48 (22%)	
Clearly stated in abstract	111 (82%)	176 (80%)	0.78
Result for primary outcome			
Benefit	65 (44%)	108 (41%)	0.65
Neutral	77 (52%)	135 (52%)	
Harm	7 (5%)	18 (7%)	
Not classifiable	0 (0%)	1 (0%)	

^a^Funding source(s) not stated for four trials

Finally, we assessed types of outcome, types of interventions and funding of trials published by individual general journals and by the specialty journals within each medical discipline. Table 4 shows the results for the general journals. Among individual general journals, industry funding was associated with 7–63% of trials. Drug interventions were evaluated in 23–74% of trials and interventions other than drugs, devices and procedures were evaluated in 6–63% of trials. The two journals that published the highest proportions of trials of drug interventions (*NEJM* and *Lancet*) also published the lowest proportion of trials of interventions other than drugs, devices and procedures. The proportion of trials with solely surrogate primary outcomes was greater than 40% for each of the general journals (range 43–87%). Conversely, in none of the journals was the proportion of trials with a hard primary outcome greater than 40% (range 7–40%).

**Table 4:** Sponsorship, interventions and primary outcome characteristics in trials published in general journals

	NEJM	JAMA	JAMA Intern Med	BMJ	Lancet	Annals Intern Med	PLoS Med
Trials, *n* Interventions, *n*	30 34	30 36	30 37	30 40	30 34	30 33	30 35
Any industry funding, *n* (%)	12 (40)	6 (20)	2 (7)*	4 (13)	19 (63)	7 (23)	3 (10)
Intervention, *n* (%)							
Drug	23 (68)	16 (44)	12 (32)	9 (23)	25 (74)	18 (55)	17 (49)
Device	0 (0)	3 (8)	0 (0)	1 (3)	4 (12)	1 (3)	0 (0)
Procedure	9 (26)	6 (17)	2 (5)	5 (13)	3 (9)	1 (3)	3 (9)
Other	2 (6)	11 (31)	23 (62)	25 (63)	2 (6)	13 (39)	15 (43)
Primary outcome, *n* (%)							
Surrogate	13 (43)	17 (57)	22 (73)	26 (87)	15 (50)	24 (80)	14 (47)
Hard	12 (40)	10 (33)	5 (17)	2 (7)	8 (27)	4 (13)	8 (27)
Mixed	5 (17)	3 (10)	3 (10)	2 (7)	7 (23)	2 (7)	8 (27)
Single	20 (67)	18 (60)	22 (73)	23 (77)	16 (53)	21 (70)	15 (50)
Composite	9 (30)	6 (20)	2 (7)	1 (3)	10 (33)	3 (10)	6 (20)
Co-primary	1 (3)	6 (20)	6 (20)	6 (20)	4 (13)	6 (20)	9 (30)

*NEJM* New England Journal of Medicine, *JAMA* Journal of the American Medical Association, *Intern Med* Internal Medicine, *BMJ* British Medical Journal, *PLoS Med* Public Library of Science Medicine

*One not stated

Table 5 shows the types of outcomes, types of interventions and funding of trials published by the specialty journals in each medical discipline. Sponsorship by industry was associated with fewer than 50% of trials published in infectious diseases and respiratory medicine journals, and over 60% of trials published in neurology, oncology and cardiology journals. More than 50% of trials in each discipline assessed drugs or devices, and only 13–22% studied interventions other than drugs, devices and procedures. The prevalence of trials with solely surrogate primary outcomes was 60–80% in all disciplines except cardiology (33%). However, 27% of trials published in the cardiology journals reported mixed surrogate-hard primary outcomes, so 60% of cardiology trials included a surrogate primary outcome.

**Table 5:** Sponsorship, interventions and primary outcome characteristics in trials published in medical specialty journals

	Neurology journals	Oncology journals	Cardiology journals	Respiratory medicine journals	Infectious diseases journals
Trials, *n* Interventions, *n*	30 33	30 32	30 32	30 32	30 38
Any industry funding, *n* (%)	21 (70)	21 (70)	19 (63)	14 (47)	11 (37)
Intervention, *n* (%)					
Drug	22 (67)	26 (78)	13 (41)	22 (69)	30 (79)
Device	1 (3)	0 (0)	4 (13)	3 (9)	0 (0)
Procedure	5 (15)	2 (6)	10 (31)	3 (9)	0 (0)
Other	5 (15)	4 (13)	5 (16)	7 (22)	8 (21)
Primary outcome, *n* (%)					
Surrogate	24 (80)	18 (60)	10 (33)	22 (73)	18 (60)
Mixed	4 (13)	5 (17)	8 (27)	0 (0)	3 (10)
Hard	2 (7)	7 (23)	12 (40)	8 (27)	9 (30)
Single	21 (70)	21 (70)	15 (50)	20 (67)	25 (83)
Composite	0 (0)	1 (3)	8 (30)	1 (3)	0 (0)
Co-primary	9 (30)	8 (27)	7 (23)	9 (30)	3 (10)

## Discussion

The majority of clinical trials published in major general medical and speciality journals in five clinical disciplines had a surrogate primary outcome; 61% of trials had only a surrogate primary outcome, and a further 15% included a surrogate outcome in a mixed primary outcome. Thus, only one quarter of the trials reported a solely hard primary outcome. Since surrogate outcomes may be unreliable in predicting hard outcomes [2, 4], our finding raises concerns that clinical trials published in these influential journals might contribute to the adoption of clinical practices that are subsequently contraindicated when trials with hard outcomes are conducted [2]. While trials with surrogate outcomes have a role in generating hypotheses about treatments and informing the design of trials with hard outcomes, their prominent publication in major journals might also have some disadvantageous effects.

The majority of trials we assessed (61%) were not sponsored by industry. Surrogate primary outcomes were present equally frequently in trials funded by industry and other sources. A higher proportion of trials published in specialty journals than general journals had industry funding. Since 86% of industry-funded trials were of either drugs or devices, compared to 44% of trials funded from non-industry sources, it is not surprising that a higher proportion of trials published in specialty journals compared to trials published in general journals were of drugs or devices. Some evidence suggests that industry-funded research disproportionately generates positive results [14, 15], but we found that the results for the primary outcome (benefit, neutral or harm) were similar in trials funded by industry or non-industry sources.

Medical devices and procedures are frequently used in medicine, and their widespread use can occur without strong evidence of efficacy and safety [10, 16]. In the current analysis, trials of devices and procedures comprised only 16% of trials published in these influential journals, while those that assessed drug interventions comprised 56%, and those that assessed interventions other than drugs, devices and procedures, 28%. The low proportion of trials evaluating devices and procedures may not reflect the importance of those interventions to clinical practice. Neither does it suggest that concerns about inadequate evidence for the efficacy and safety of devices are being addressed [10, 12, 17]. The importance of evaluating procedures is underscored by a meta-analysis of placebo-controlled trials of surgical interventions, which identified only 53 trials, in half of which the procedure was not superior to a placebo [18].

General medical journals varied widely in the proportions of trials they published that had any industry funding (7–63%) and of the types of intervention assessed. These characteristics are likely co-dependent, as industry funding is more likely to support trials of drugs and devices than trials of health-care delivery or health behaviours. Thus, the two general journals that published the highest proportion of industry-funded trials also published the highest proportion of trials of drugs and devices. Consistently, the proportion of trials with a hard primary outcome published in each of these influential general journals was <30%. Among the specialty journals, although there was also some variability in the proportions of published trials with industry funding, it was in each case more than 33%. With the exception of cardiology, journals in each discipline were strikingly similar in publishing mainly trials of drug interventions (67–79%). Few published trials in each discipline (13–22%) were of interventions other than drugs, devices or procedures. For four of five disciplines, trials with hard outcomes represented <30% of published trials, and in all disciplines such trials were the minority of published trials.

Our study has several limitations. Some trials were reported to have both industry and non-industry funding, but we were unable to ascertain whether one source predominated. Some non-industry-funding sources might derive income from commercial sources, but we could not determine this. We reported the characteristics of published trials but could not ascertain whether the results we observed reflect the characteristics of trials submitted for journal review; nonetheless our findings reflect the set of trial results most visible to practitioners. Categorising outcomes as surrogate or hard can sometimes be difficult [19], but we made independent assessments and recorded the majority opinion in the case of disagreement. Our analysis was confined to results published over 2 years, so does not permit inference about temporal patterns of trial publishing. We assessed the primary outcome in the trial publication rather than that in the trial registration document. We categorised trial results according to reported statistical significance, which might not reflect clinical importance. We did not undertake an assessment of risk of bias in the included trials, so cannot determine whether trial quality is associated with the variables we assessed.

## Conclusion

The majority of trials published in high-impact general and specialty medical journals have surrogate primary outcomes and assess drug interventions. Trials of devices and procedures are infrequently published in such journals, despite the prominence of each type of intervention in clinical practice. Industry funding is more common for trials published in specialty than general journals but is not associated with more positive results for trial primary outcomes or with a greater preponderance of surrogate outcomes.
